# The Maxi-K (BK) Channel Antagonist Penitrem A as a Novel Breast Cancer-Targeted Therapeutic

**DOI:** 10.3390/md16050157

**Published:** 2018-05-11

**Authors:** Amira A. Goda, Abu Bakar Siddique, Mohamed Mohyeldin, Nehad M. Ayoub, Khalid A. El Sayed

**Affiliations:** 1 Department of Basic Pharmaceutical Sciences, School of Pharmacy, University of Louisiana at Monroe, 1800 Bienville Drive, Monroe, LA 71201, USA; amirakareem16@gmail.com (A.A.G.); siddiqab@warhawks.ulm.edu (A.B.S.); Mohamed.mohyeldin@alexu.edu.eg (M.M.); 2 Department of Clinical Pharmacy, Faculty of Pharmacy, Jordan University of Science and Technology, Irbid 22110, Jordan; nmayoub@just.edu.jo; 3 Department of Pharmacognosy, Faculty of Pharmacy, Alexandria University, Alexandria 21521, Egypt

**Keywords:** breast cancer, BK (Maxi-K) channel, EGFR, HER2, penitrem A, gefitinib, lapatinib, TNF-α

## Abstract

Breast cancer (BC) is a heterogeneous disease with different molecular subtypes. The high conductance calcium-activated potassium channels (BK, Maxi-K channels) play an important role in the survival of some BC phenotypes, via membrane hyperpolarization and regulation of cell cycle. BK channels have been implicated in BC cell proliferation and invasion. Penitrems are indole diterpene alkaloids produced by various terrestrial and marine *Penicillium* species. Penitrem A (**1**) is a selective BK channel antagonist with reported antiproliferative and anti-invasive activities against multiple malignancies, including BC. This study reports the high expression of BK channel in different BC subtypes. In silico BK channel binding affinity correlates with the antiproliferative activities of selected penitrem analogs. **1** showed the best binding fitting at multiple BK channel crystal structures, targeting the calcium-sensing aspartic acid moieties at the calcium bowel and calcium binding sites. Further, **1** reduced the levels of BK channel expression and increased expression of TNF-α in different BC cell types. Penitrem A (**1**) induced G1 cell cycle arrest of BC cells, and induced upregulation of the arrest protein p27. Combination treatment of **1** with targeted anti-HER drugs resulted in synergistic antiproliferative activity, which was associated with reduced EGFR and HER2 receptor activation, as well as reduced active forms of AKT and STAT3. Collectively, the BK channel antagonists represented by penitrem A can be novel sensitizing, chemotherapeutics synergizing, and therapeutic agents for targeted BC therapy.

## 1. Introduction

Breast cancer (BC) is the most commonly diagnosed malignancy among women, globally contributing to high mortality rates [1]. BC is highly heterogeneous at both molecular and clinical level, with various pathological and molecular subtypes [2]. Four major molecular subtypes of BC were identified; these include luminal A, luminal B, human epidermal growth factor receptor 2 (HER2)-positive, and basal-like subtypes [3,4,5]. Both luminal A and luminal B tumors are hormone receptor-positive, and have expression patterns reminiscent of the luminal epithelial component of the breast [6]. HER2-enriched tumors are characterized by overexpression/amplification of the ErbB2/HER2 gene, and are generally hormone receptor-negative [2,7]. Basal-like tumors are predominantly triple-negative, lacking the expression of hormone receptors and HER2 [7]. The subtypes are associated with distinct pathological features and clinical outcomes [2].

The Maxi-K channels, also known as BK/BKCa/Slo1/KCa1.1 channels, are characterized by ubiquitous tissue expression and large conductance to potassium ions [8]. The channel is sensitive to voltage and Ca^2+^. BK channels are essential for smooth muscle contraction, neurotransmitter release, hormone secretion, and gene expression [9]. In addition, BK channels have been shown to associate with multiplicity of plasma membranes and intracellular proteins as linkers of membrane potential, cell metabolism, and cell signaling [8]. The structure of BK channel is composed of four Slo1 subunits. Each Slo1 subunit consists of four α-subunits and four β-subunits [10,11,12]. Each α-subunit is composed of seven putative transmembrane-spanning α-helical segments (pore-gate domain, PGD). It controls ions conductance, selectivity, and voltage change sensing [10,11,12]. The cytoplasmic carboxy tail of the α-subunit possesses two intrinsic high-affinity Ca^2+^ binding and phosphorylation sites, known to direct the gating mechanism (cytosolic tail domain, CTD). Each β-subunit is formed of two transmembrane domains with a long extracellular linker, and both its amino- and carboxy-terminals are cytoplasmic (voltage sensor domain, VSD) [10,11,12]. Dysregulation or overexpression of BK channels have been associated with altered cell cycle progression [13], cell proliferation [14,15,16,17,18,19,20], and migration. These features are fundamental for cancer development and progression [21,22,23,24]. In this regard, earlier studies indicated variable levels of BK channel expression in human BC cells [25]. In addition, electrophysiological studies on cervical and BC cells suggested that BK channels are directly activated by estrogens, which could have an essential role in uterus, breast, and prostate cancers [26,27]. Taken together, pharmacological blockade of BK channels would be expected to suppress cancer cell proliferation [13].

**Scheme 1:**
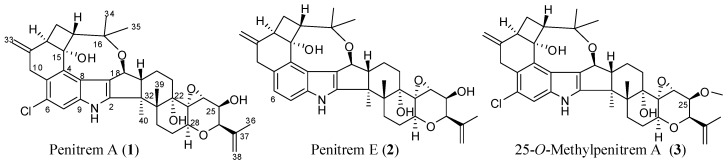
Chemical structures of tested penitrems **1**–**3**.

Penitrems are indole diterpene neurotoxic alkaloids produced by various terrestrial and marine *Penicillium* species [28,29]. This study team reported penitrems **1**, **2** (Scheme 1), and others from a marine-derived *Penicillium commune* isolate GS20 isolated from sponge and sediment samples collected in the Arabian Gulf [30,31]. Penitrems have potent tremorgenic activity in mammals, secondary to the antagonism of BK channels [28,29]. Previous findings from our laboratory revealed the potential anticancer effects of penitrems as inhibitors of proliferation, migration, and invasion of BC cells [30,31]. The mechanism for these reported anticancer effects was associated with the suppression of the Wnt/β-catenin pathway in BC cells [30].

In this study, penitrems were applied in terms of BK channel inhibitors, to assess their antiproliferative effects in multiple BC cell lines, in vitro. The antiproliferative activity of the most potent **1** was assessed individually, and in combination with targeted therapy. The study also compares the in silico binding mode of **1** at multiple BK channel crystal structures with its related less active analogs, **2** and **3** (Scheme 1).

## 2. Results

### 2.1. Antiproliferative Effects of Penitrems in Breast Cancer Cells In Vitro

**Figure 1:**
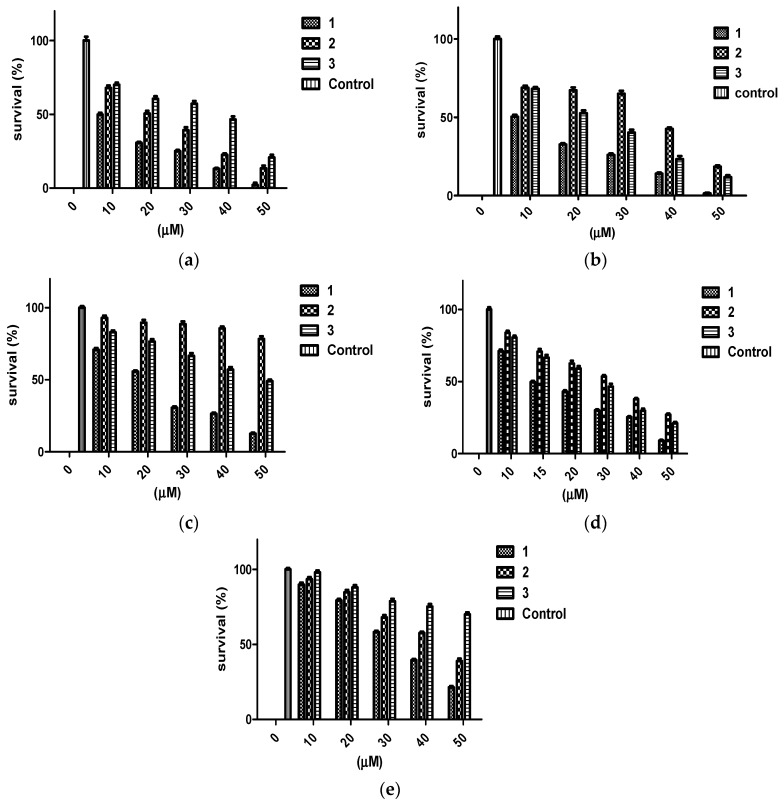
Antiproliferative effects of penitrems **1**–**3** against BC and non-tumorigenic cells. Viability of (**a**) BT-474, (**b**) MDA-MB-231, (**c**) SK-BR-3, (**d**) CRL-2765, and (**e**) MCF-12A cells after incubation with increasing concentrations of **1**–**3** for 48 h in cell cultures. All values are relative to vehicle control (100% survival). Bars represent mean ± SEM for four replicates within each group.

The antiproliferative activity of penitrems was assessed using MTT cell viability assay. Multiple human BC cell lines representing the different molecular subtypes were tested, including MDA-MB-231, BT-474, and SK-BR-3 cells, along with the human neuronal Schwann cells CRL-2765 and the non-tumorigenic mammary epithelial MCF-12A cells. Penitrem A (**1**) resulted in a dose-dependent inhibition among all three tested BC cell lines after 48 h culture duration (Figure 1). Among BC cell lines exposed to **1**, the triple-negative MDA-MB-231 cells were most sensitive to the antiproliferative effects of **1**, as indicated by lowest IC_50_ value (Table 1). Penitrem E (**2**) and 25-*O*-methylpenitrem A (**3**) showed less inhibition of BC cell growth compared to **1**. MDA-MB-231 cells were the most sensitive to growth suppression by **2**, while the HER2-positive SK-BR-3 cells were most inhibited by **3**. With respect to non-tumorigenic cells, **1** was the most toxic, with IC_50_ values of 33.7 µM in MCF-12A (non-tumorigenic human mammary epithelial cells), and 22.6 µM in Schwann C RL-2765 (peripheral neuronal cells), respectively (Figure 1, Table 1).

### 2.2. In Silico Binding of Penitrems with BK Channel

Both human BK channel PDB crystal structures, 3NAF and 3MT5, were used in this study for molecular docking [32,33,34]. Penitrems **1**–**3** were virtually screened for their ability to bind the BK channel crystal structures 3NAF and 3MT5, to correlate their binding modes and affinity with the antiproliferative activity of the compounds. Binding of the intracellular Ca^2+^ at the CTD enhances the BK channel opening. Each Slo1 subunit CTD has two Ca^2+^ high affinity binding sites; the RCK1, which includes the side-chain carboxylates of Asp367 and Glu535, as well as the main chain carbonyl of Arg514, in addition to the C-terminus of RCK2 domain, with a string of aspartic residues at the calcium bowel [35]. In the calcium bowel, the side chain carboxylate groups of Asp895 and Asp897 provide direct coordinates to bind Ca^2+^ ion, consistent with previous mutagenesis and biochemistry literature [36]. The side chain of Asp894 does not directly contact with Ca^2+^, but instead, it forms salt bridges with Arg1018 and Lys1030. The N-terminus of the RCK1 domain identified two acidic residues, GLUlu374 and Glu399, which are close to the voltage sensor activation site Asp99 and Asn172 at the edge of VSD.

**Figure 2:**
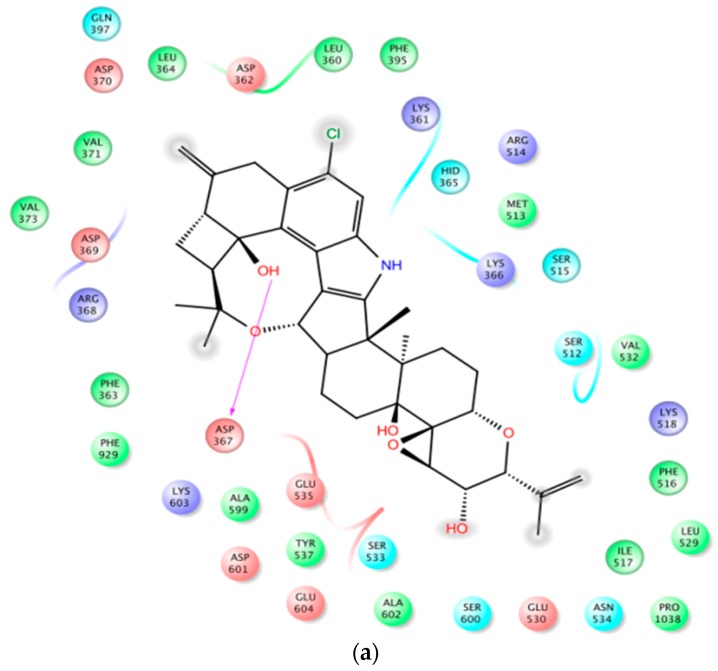

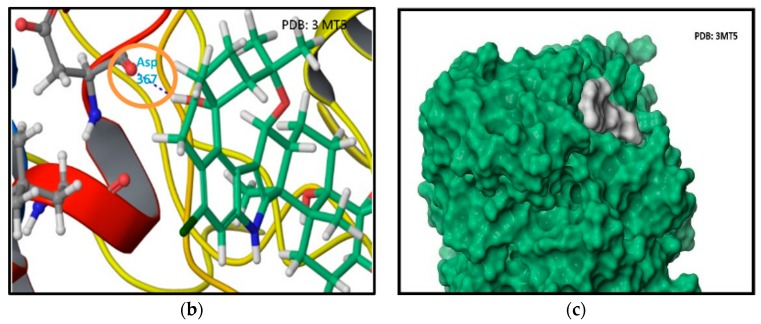
Binding mode of **1** at the Ca^2+^ binding site of BK channel crystal structure PDB 3MT5. (**a**) The 2D binding mode of **1** at the Ca^2+^ binding site. Its C-15 hydroxyl group contributed hydrogen bonding donor interaction with Asp367, which has high affinity for the Ca^2+^ binding. (**b**) The overlay of 3D structure of **1** at the Ca^2+^ binding site of the PDB 3MT5. (**c**) Shape fitting of **1** within the Ca^2+^ binding site of BK PDB structure 3MT5.

**Figure 3:**
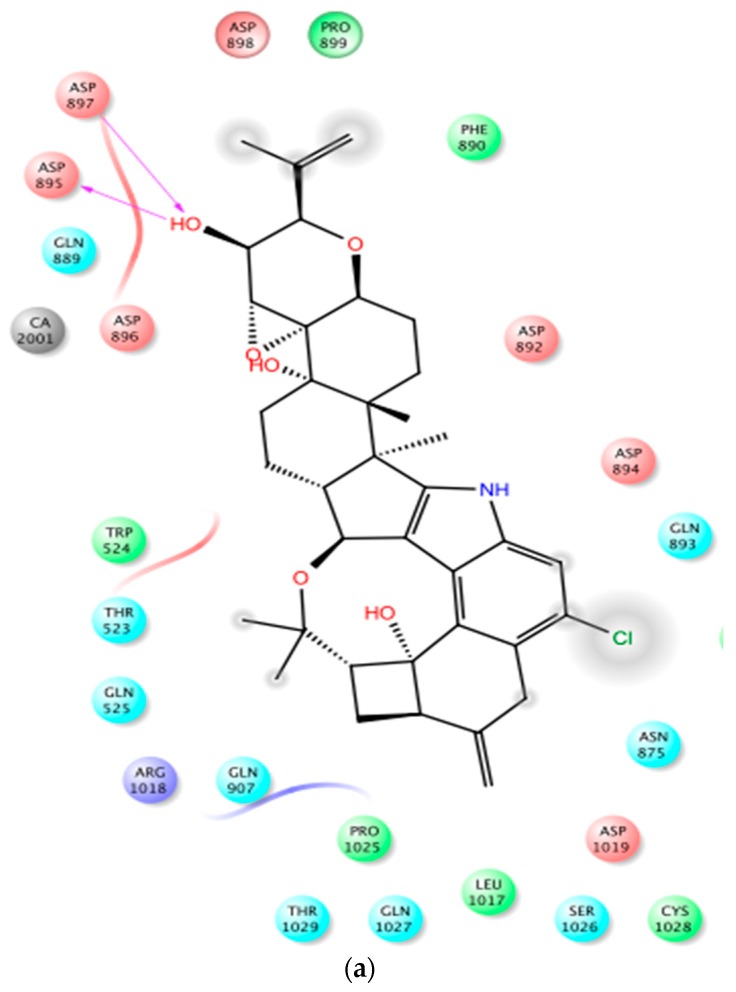

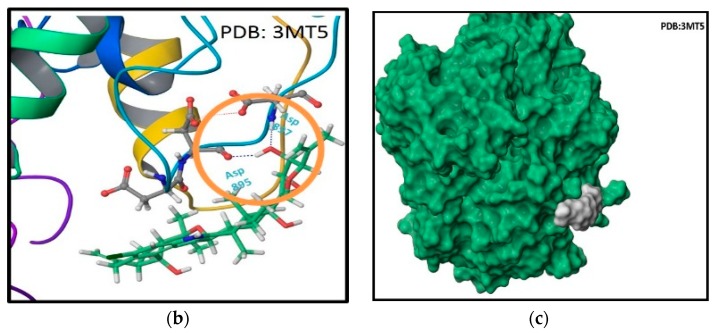
Binding mode of **1** at the Ca^2+^ binding bowel of the BK channel crystal structure PDB 3MT5. (**a**) The 2D binding mode of **1** at the Ca^2+^ binding bowel. Its C-25 hydroxyl group contributed hydrogen bonding donor interaction with ASP895 and accepted hydrogen bonding interaction with Asp897. Both aspartic acid moieties are known to provide direct coordinate for Ca^2+^ binding at the Ca^2+^ bowel site. (**b**) The overlay of 3D structure of **1** at the Ca^2+^ binding bowel of the PDB 3MT5. (**c**) Shape fitting of **1** within the Ca^2+^ binding bowel pocket of BK channel PDB structure 3MT5.

**Figure 4:**
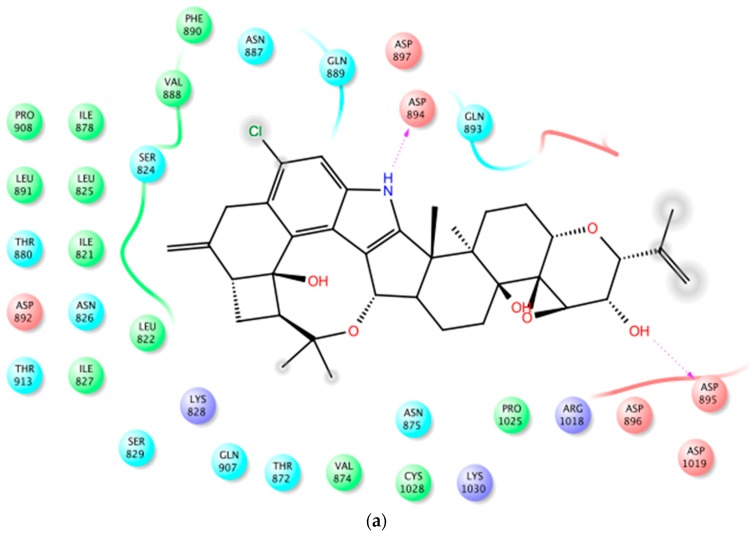

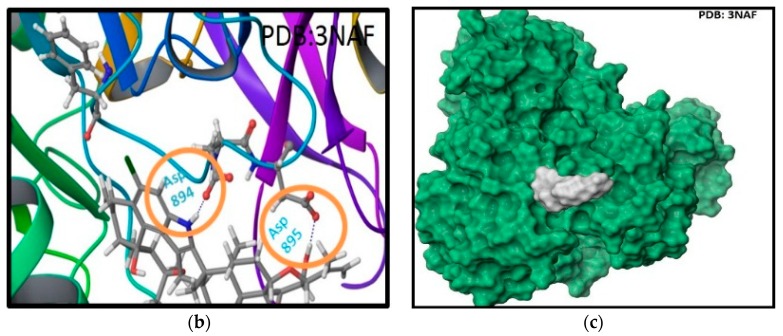
Binding mode of **1** at the BK channel crystal structure PDB 3NAF. (**a**) The 2D binding mode of **1** at the Ca^2+^ binding bowel of PDB 3NAF. NH-1 of **1** contributed hydrogen bonding donor interaction with Asp894 while the C-25 hydroxyl contributed hydrogen bonding donor interaction with Asp895 at the calcium bowel site. These aspartic acid moieties provide direct coordinate for Ca^2+^ binding at the Ca^2+^ bowel site. (**b**) The overlay of 3D structure of **1** at the Ca^2+^ binding bowel of the PDB 3NAF. (**c**) Shape fitting of **1** within the Ca^2+^ binding bowel pocket PDB structure 3NAF.

In PDB 3MT5, the C-15 tertiary alcohol group of **1** contributed a hydrogen bonding donor interaction with Asp367, which has high affinity for Ca^2+^ binding (Figure 2). Similarly, the C-25 secondary alcohol of **1** contributed hydrogen bonding donor interaction with Asp895, and accepted hydrogen bonding interaction with Asp897 at the Ca^2+^ bowel site, which provides direct coordinate to bind Ca^2+^ (Figure 3). In PDB structure 3NAF, the NH-1 of **1** contributed hydrogen bonding donor interaction with Asp894 and the C-25 hydroxyl group contributed hydrogen bonding donor interaction with Asp895 at the Ca^2+^ bowel site, justifying its antagonistic activity to BK channel (Figure 4). Meanwhile, penitrem analog **3** showed only two possible interactions at the calcium bowel of PDB 3MT5 (Figure S1). Its C-15 tertiary hydroxyl group showed hydrogen bonding donor interaction with Glu521, while its NH-1 showed hydrogen bonding donor interaction with Gln525.

**Figure 5:**
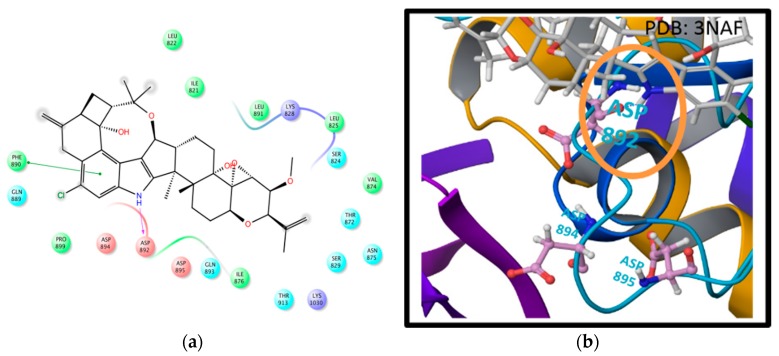
Binding mode of **3** at the Ca^2+^ bowel of the PDB crystal structure 3NAF. (**a**) The 2D binding mode of **3** at the Ca^2+^ bowel showing its NH-1 hydrogen bonding donor interaction with the critical Asp892. (**b**) The overlay of 3D structure of **3** at the calcium bowel of the PDB 3NAF crystal structure.

There were no interactions with the essential aspartates at the calcium bowel, which justify its weak BK channel inhibitory activity. The only important interaction for **3** in the calcium bowel of PDB 3NAF was a hydrogen bonding donor interaction between its NH-1 with Asp892 (Figure 5). Penitrem **2** contributed only a hydrogen bonding donor interaction via its NH-1 with Phe890 at the calcium bowel of PDB 3NAF, and showed no interactions with PDB 3MT5 (Figures S2 and S3).

### 2.3. Expression of BK Channels in BC Cells and In Vitro Impact of Penitrems on Channel Expression

**Figure 6:**
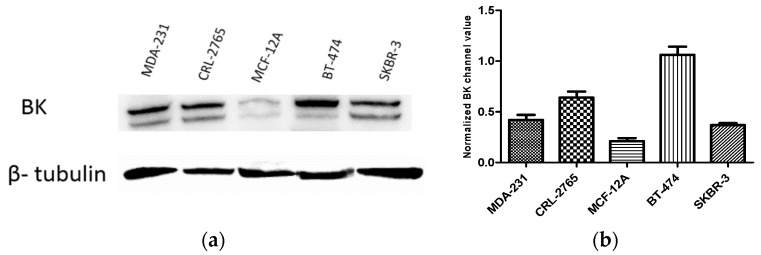
Expression of BK channel subunits α-1 (KCNMA1) in BC and non-tumorigenic cell lines. (**a**) Western blots representing total levels of the BK channel subunits α-1 (KCNMA1) in the different cell lines. (**b**) Bar graphs indicating densitometric quantitative analysis performed on all blots, in which optical density of each band was normalized with corresponding β-tubulin.

The basic functional unit of BK channels is the tetramer of the pore-forming α-subunits (KCa1.1 or Slo1) encoded by the gene KCNMA1 [37,38,39]. The expression levels of BK channel subunits α-1 (KCNMA1) in multiple BC cell lines was compared with the neuronal Schwann cells, CRL-2765, and the non-tumorigenic mammary epithelial MCF-12A cells using Western blot analysis (Figure 6).

**Figure 7:**
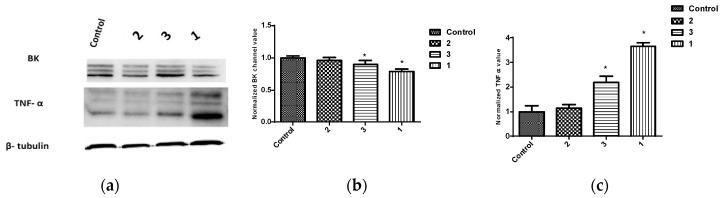
In vitro effects of 10 µM treatments of penitrems **1**–**3** on the expression of BK channel (KCNMA1) and TNF-α (D2D4) in MDA-MB-231 cells using Western blot analysis. (**a**) Western blots for cells treated with penitrems **1**–**3**. (**b**) Western blot quantification of the in vitro effects of penitrem **1**–**3** treatments on the expression of KCNMA1. (**c**) Western blotting quantification of the effects of **1**–**3** treatments on the activation of TNF-α. Vertical bars indicate the normalized protein value ± SEM. *: indicate significant differences (*p* ≤ 0.05).

**Figure 8:**
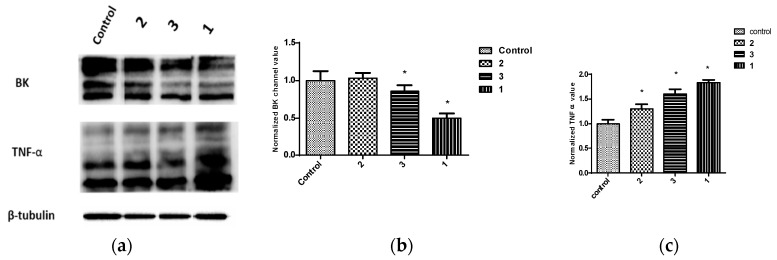
In vitro effects of 10 µM treatments of penitrems **1**–**3** on the expression of BK channel (KCNMA1) and activation of TNF-α (D2D4) in BT-474 cells using Western blot analysis. (**a**) Western blot for cells treated with penitrems **1**–**3**. (**b**) Western blot quantification of the in vitro effects of penitrems **1**–**3** treatment on the expression of KCNMA1. (**c**) Western blot quantification of the effects of penitrems **1**–**3** treatment on the activation of TNF-α. Vertical bars indicate the normalized protein value ± SEM. *: indicate significant differences (*p* ≤ 0.05).

**Figure 9:**
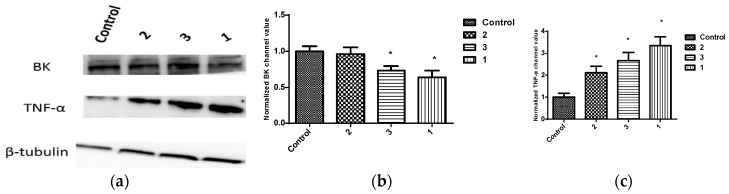
In vitro effects of 10 µM treatments of penitrems **1**–**3** on the expression of BK channel (KCNMA1) and activation of TNF-α (D2D4) in SK-BR-3 cells using Western blot analysis. (**a**) Western blot for cells treated with penitrems **1**–**3**. (**b**) Western blotting quantification of the in vitro effects of penitrem **1**–**3** treatments on the expression of KCNMA1. (**c**) Western blot quantification of the effects of **1**–**3** treatments on the activation of TNF-α. Vertical bars indicate the normalized protein value ± SEM. *: indicate significant differences (*p* ≤ 0.05).

The effect of penitrems on the levels of KCNMA1 and TNF-α, a marker for BK antagonism [40,41,42,43,44,45] were assessed in BC cell lines (Figure 7, Figure 8 and Figure 9). Consistent with its antiproliferative activity, **1** resulted in the greatest reduction in the total levels of BK channel subunits α-1 (KCNMA1), an effect which was also associated with increased total levels of TNF-α among BC cell lines (Figure 7, Figure 8 and Figure 9). Similar effects were observed with 25-*O*-methylpenitrem A (**3**), however, to a lesser extent than **1**. Penitrem **2** treatment did not cause changes to the total levels of KCNMA1 in BC cells. Compound **2**, however, increased total levels of TNF-α in both BT-474 and SK-BR-3 cells compared to control groups (Figure 7, Figure 8 and Figure 9).

**Figure 10:**
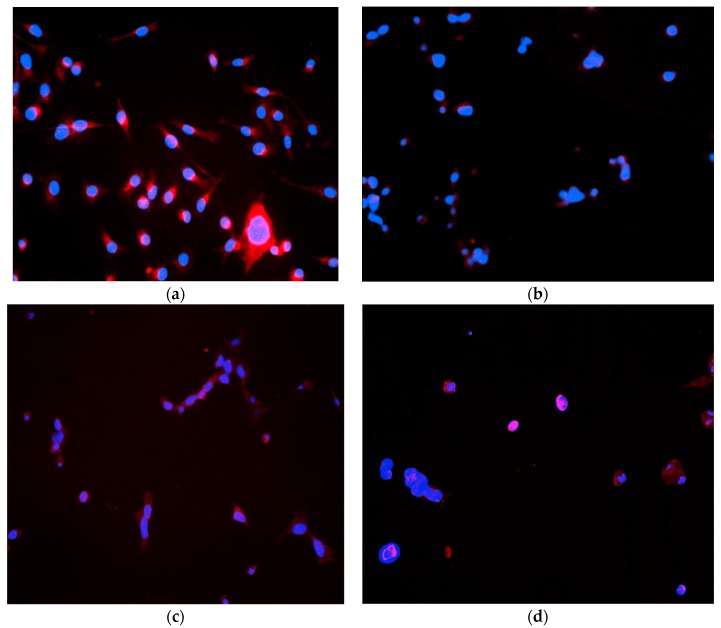
Immunocytochemical fluorescence staining of the total levels of BK channel subunits α-1 (KCNMA1) in MDA-MB-231 and BT-474 BC cells treated with **1** at its IC_50_ concentration, 9.8 and 10.3 µM, respectively, for 24 h. (**a**) MDA-MB-231 cells treated with vehicle control. (**b**) MDA-MB-231 cells treated with **1** at 9.8 µM. (**c**) BT-474 cells treated with vehicle control. (**d**) BT-474 cells treated with **1** at 10.3 µM. Red staining indicates positive immunofluorescence signal for KCNMA1 and blue staining indicates cell nuclei counter-stained with DAPI. Magnification of each photomicrograph is 20×. (**a**) or treatment media containing 1 treatment for 24 h (**b**). Cells were then fixed in pre-cooled acetone and subjected to immunofluorescence analysis for detection of total KCNMA1 level.

In the same context, immunofluorescent staining of MDA-MB-231 (Figure 10a,b) and BT-474 cells (Figure 10c,d) indicated strong cytoplasmic expression of KCNMA1 in vehicle-treated culture media (Figure 10a,c). Penitrem **1** treatment caused significant reduction in the total level of KCNMA1 compared to cells in vehicle-treated control groups (Figure 10a,c). Penitrem **1** treatments caused remarkable reduction in the total levels of KCNMA1 in both cell lines compared to cells of vehicle-treated controls (Figure 10b,d).

### 2.4. Effect of Penitrem A Treatment on Cell Cycle Progression in BC Cells

**Figure 11:**
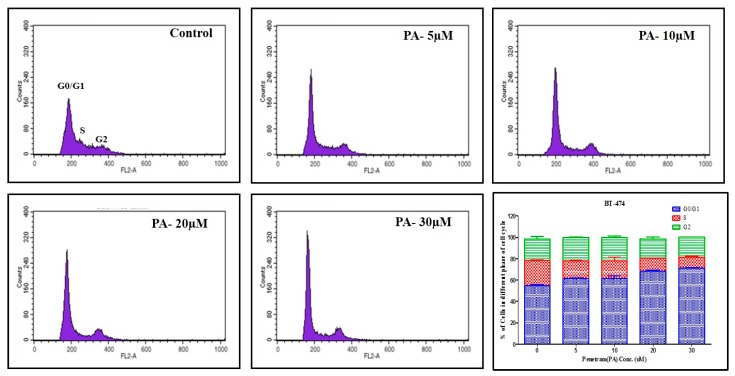
Flow cytometry analysis for cell cycle progression in BT-474 cells treated with **1** (PA). Cells in the various treatment groups were synchronized in G1 phase. Histograms were generated using Cell Quest software (PI staining). Column graph shows percentage of BT-474 cells in each phase of the cell cycle. Vertical bars show the average percentage of three independent experiments.

The effect of **1** on cell cycle progression of BT-474 cells was evaluated using flow cytometry by applying propidium iodide (PI) staining (Figure 11) [13,45,46,47,48]. BT-474 cells were treated with various doses of **1** for 48 h, prior to fixation and staining. BT-474 cells exposed to various concentrations of **1** resulted in a dose-dependent increase (53% to 70%) in the proportion of cells in G1 phase compared to cells in vehicle-treated control group, with the maximal effect observed at 30 μM.

### 2.5. Effects of Combined Treatment of Targeted Agents and Penitrem A on the Growth of BC Cells

Penitrem **1** is known to be a potent BK channels antagonist at very low molar concentration [49,50]. Therefore, subeffective dose combinations of **1** with either lapatinib (LP) or gefitinib (GF) were hypothesized to have a superior antiproliferative activity, compared to individual treatments in BC cells.

**Figure 12:**
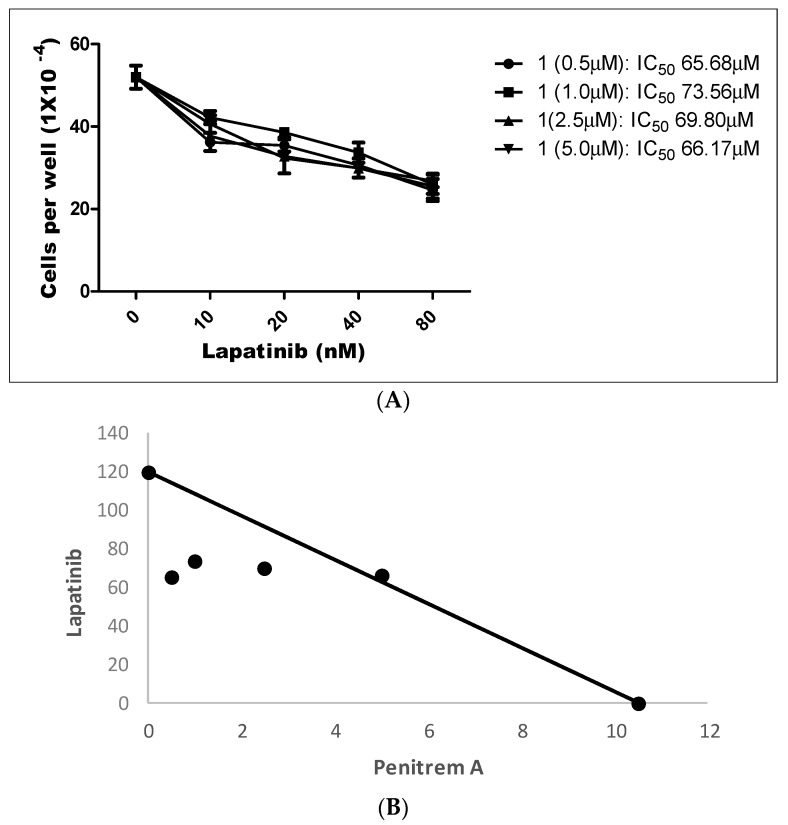
(**A**) Effects of combined treatment of penitrem **1** and LP on BT-474 cell viability after 48 h culture period. Bars represent mean ± SEM for four replicates within each group. (**B**) Isobologram of penitrem A (**1**, µM) and LP (nM) antiproliferative effect in BT-474 cells. IC_50_ concentrations for **1** and LP were plotted on the *X*- and *Y*-axis, respectively. The solid line connecting these points represents the concentration of each compound required to induce the same relative growth inhibition when used in combination, if the interaction between the compounds is additive. Each data point on the isobologram represents the actual concentrations of **1** and LP which induced 50% inhibition of cell growth when used in combination.

LP treatment inhibited the growth of BT-474 cells in a dose-dependent manner, with an IC_50_ value of 123.0 nM (data not shown). Penitrem **1** has been shown to inhibit growth of BT-474 cells at an IC_50_ value of 10.3 μM (Table 1). Figure 12 shows the effect of a combined range of subeffective concentrations of LP (10–80 nM) with a range of **1** concentrations (0.5, 1.0, 2.5, and 5.0 µM). Combined treatment of LP and **1** resulted in dose-dependent inhibition of BT-474 cell proliferation after 48 h in culture (Figure 12A). The combination of LP with **1** effectively reduced the IC_50_ concentration of LP within each treatment combination, compared to individual monotherapy treatments (Table 2).

To further evaluate the nature of interaction between both compounds, combination index (CI) and isobologram analysis were conducted. CI is a quantitative representation of pharmacological interaction between two drugs, in which values <1, 1, and >1 indicate synergistic, additive, and antagonistic effects, respectively [51,52]. CI values are calculated as follows: CI = [Xc/X + Tc/T], where X and T stand for the concentrations of individual combination ingredients that induce 50% cell growth inhibition (IC_50_); Xc and Tc are the concentrations of combination ingredients that induce 50% cell growth inhibition when used in combination as determined by non-linear regression curve fit analysis [51,52]. Isobologram analysis is a graphical method used to evaluate the effect of equally effective dose pairs for a single effect level [53]. Calculated CI of **1**-LP combined treatments indicated synergism with values of 0.58, 0.70, and 0.81, respectively, for the first three treatments (Figure 12A,B and Table 2). The use of combined 5.0 μM of **1** with LP afforded an IC_50_ of 66.17 nM and CI of 1.03, a very weakly antagonistic combination (Figure 12A,B and Table 2). In addition, isobologram showed synergistic interaction between both compounds as indicated by three combinations of data points located below the line of additive activity (Figure 12B).

**Figure 13:**
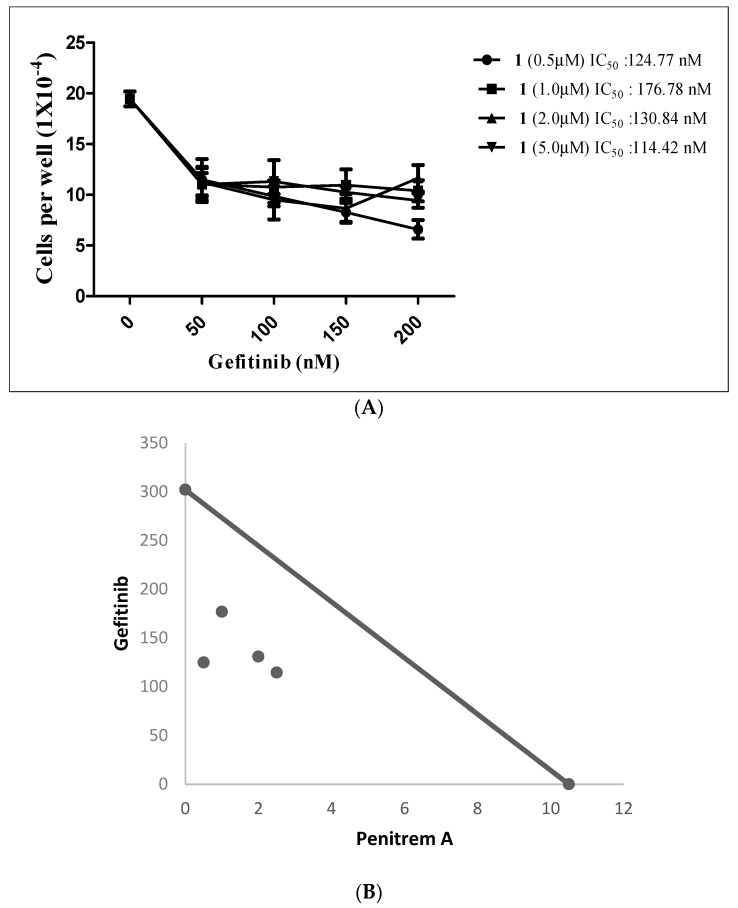
(**A**) Effects of combined subeffective doses of **1** and GF in BT-474 cells after 48 h culture period. Bars represent mean ± SEM for four replicates within each group. (**B**) Isobologram of **1** (µM) and GF (nM) antiproliferative effect in BT-474 cells. IC_50_ concentrations for **1** and GF were plotted on the *X*- and *Y*-axis, respectively. The solid line connecting these points represents the concentration of each compound required to induce the same relative growth inhibition when used in combination, if the interaction between the compounds is additive. Each data point on the isobologram represents the actual concentrations of **1** and GF which induced 50% inhibition of cell growth when used in combination.

Gefitinib (GF), an EGFR small molecule inhibitor, suppressed the growth of BT-474 cells in a dose-dependent fashion, with IC_50_ value of 302.4 nM (data not shown). Figure 13A shows the effects of combined subeffective concentrations of GF (50–200 nM) with range of **1** concentrations (0.5, 1.0, 2.5, and 5.0 µM). The combination of both GF and **1** suppressed the growth of BT-474 cells in a dose-dependent manner (Figure 13A). The combination of GF with **1** effectively reduced the GF IC_50_ concentrations for each tested treatment combination, compared to single GF treatment (Table 2). Calculated CI of **1**-GF combined treatments indicated synergism, with values of 0.46, 0.68, 0.68, and 0.87, respectively (Figure 13A,B and Table 2). Isobologram analysis showed synergistic interaction between both compounds, as indicated by four data points located below line of additive activity (Figure 13B).

### 2.6. Effects of Combined Treatment of Targeted Agents and Penitrem A on Receptor Tyrosine Kinases (RTKs) and Their Downstream Effectors

**Figure 14:**
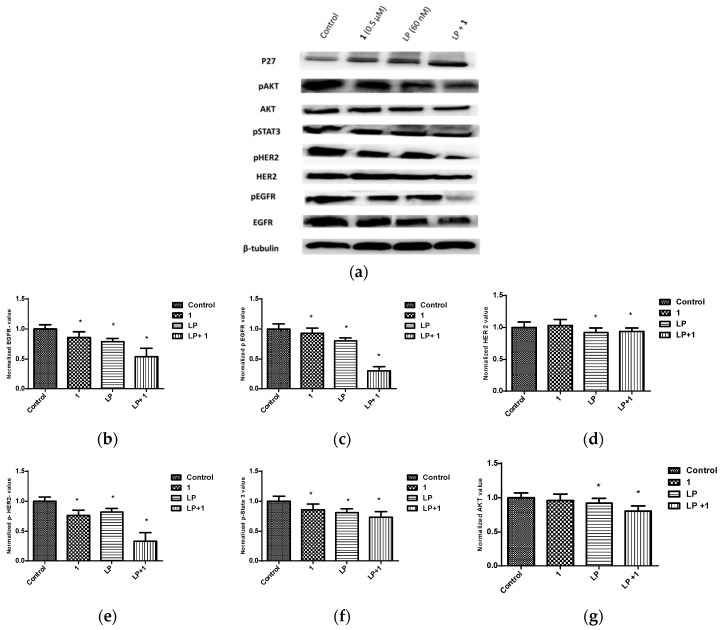

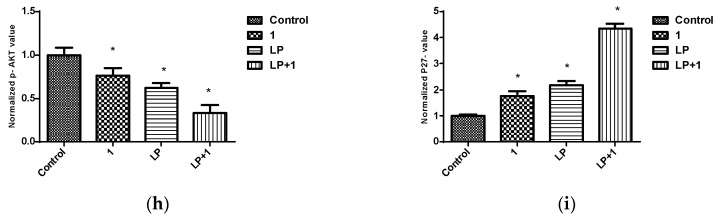
Effects of combined treatments of **1** and LP on RTKs and downstream effectors in BT-474 cells. (**a**) Western blots for BT-474 cells treated with vehicle control, **1** (0.5 μM), LP (60 nM), and **1**-LP combination. (**b**–**i**) Quantification analysis for Western blots for the effect of treatment on levels of EGFR, p-EGFR, HER2, p-HER2, p-STAT3, AKT, p-AKT, and p27, respectively. Vertical bars in the graphs indicate tubulin normalized protein value ± SEM. *: indicate significant differences (*p* ≤ 0.05).

Earlier studies showed that BK channels regulate Ca^2+^ signaling, which in turn mediates EGFR transactivation [54]. In this part, the effect of **1** and combined targeted therapy on total and active levels of RTKs, along with their downstream transducers, was evaluated using Western blot analysis. The concentrations of 0.5 µM of **1** and 60 nM LP, which resulted in synergistic antiproliferative activity in viability studies in vitro, were further applied to assess combination effects on downstream pathways. This combined LP and **1** treatment reduced the phosphorylated (active) levels of EGFR, HER2, and AKT, compared to individual treatments and vehicle-treated controls (Figure 14). However, total levels of EGFR and HER2 receptors did not remarkably change in response to treatment. In addition, there was an increase in the levels of the arrest protein p27 with combined treatment. Combination of **1**-LP mediated the inhibition of AKT signaling, leading to p27 stabilization and accumulation, which is a known inhibitor of CDK2. This resulted in G_1_ arrest and subsequent growth inhibition in BT-474 cells [51,55].

**Figure 15:**
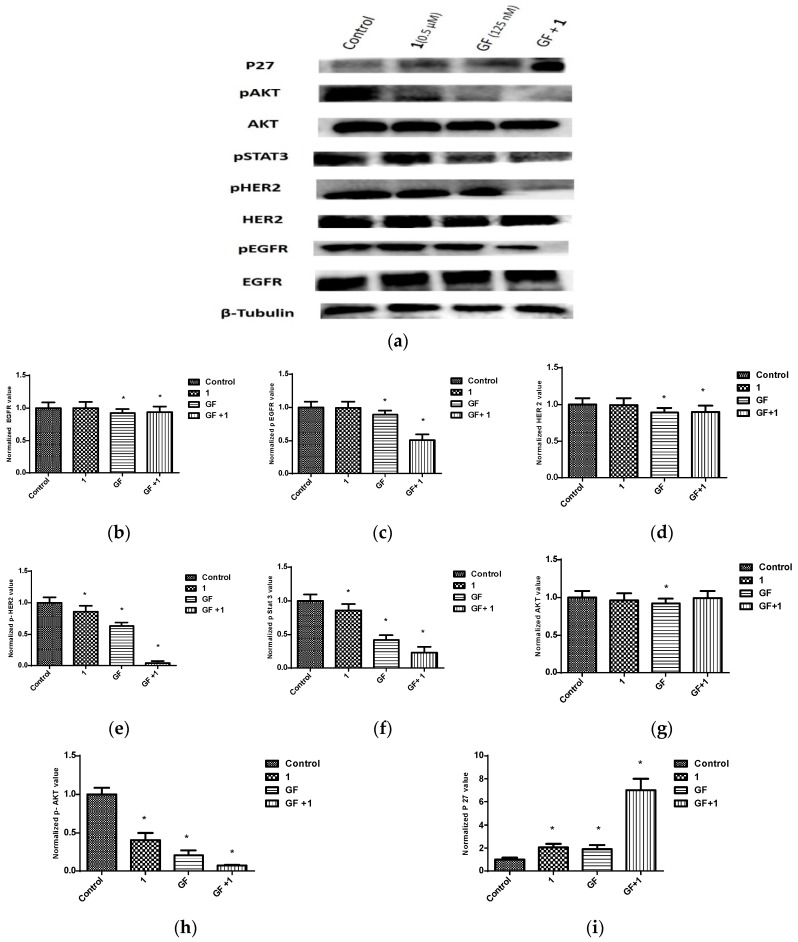
Effects of combined treatments of **1** and GF on RTK and downstream effectors in BT-474 cells. (**a**) Western blots for BT-474 cells treated with vehicle control, **1** (0.5 μM), GF (123 nM), and **1**-GF combination. (**b**–**i**) Quantification analysis for Western blots for the effect of treatment on levels of EGFR, p-EGFR, HER2, p-HER2, p-STAT3, AKT, p-AKT, and p27, respectively. Vertical bars in the graphs indicate tubulin normalized protein value ± SEM. *: indicate significant differences (*p* ≤ 0.05).

Similarly, the synergistic combination of **1** at 0.5 µM with GF 125 nM caused a reduction in the levels of p-EGFR, p-HER2, p-AKT, and p-STAT3 (Figure 15). In addition, this combination upregulated the expression of the arrest protein p27.

## 3. Discussion

Three penitrem alkaloids, including **1** and **2**, previously isolated from the marine derived *P*. *commune* isolate GS20 [30,31], along with the semisynthetic **3**, were selected for testing their antiproliferative effects in BC cell lines in vitro [30,32]. This selection represents our previously reported pharmacophoric map of penitrems for anticancer activity [30,31]. Penitrem **1** represents the original natural parent of penitrems, while **2** is missing the important C-6 chloro substitution. The 25-*O*-methylated analog **3** is a semisynthetic product in which the free C-25 secondary alcohol pharmacophore was blocked by methylation.

Various K^+^ channels are involved in the control of Ca^2+^ homeostasis, and hence, deregulation of their expression and/or activity will significantly affect cell proliferation [33]. Clearly, **1** is the most potent BK channel antagonist, and is associated with most remarkable antiproliferative effects among different BC cell lines, compared to other tested penitrems **2** and **3**. While growth inhibition of MDA-MB-231 and BT-474 with **1** was comparable, higher concentration of **1** was reported for the inhibition of SK-BR-3 cell growth. Although potentially toxic to non-tumorigenic cells, the IC_50_ concentrations of **1** needed to suppress growth of both MCF-12A mammary epithelium and neuronal cells were multiple-fold higher than those required to inhibit BC cell growth (Table 1).

Penitrem **1** showed good fitting within the Ca^2+^ binding and Ca^2+^ bowel sites (Figure 2, Figure 3 and Figure 4) in crystal structures 3MT5 and 3NAF, respectively, unlike **2** and **3**.

Among BC cell lines, highest expression levels were observed in the luminal B BT-474 cells. MDA-MB-231 and SK-BR-3 cells showed comparable levels KCNMA1. The lowest expression level of KCNMA1 was observed however in non-tumorigenic MCF-12A mammary epithelial cells (Figure 6).

TNF-α is a prototype cytokine which imparts a multitude of actions on many cell types, including neurons [40]. TNF-α was originally discovered as a serum factor causing necrosis of tumor cells [41]. TNF-α production in human macrophages is dependent on Ca^2+^/calmodulin/calmodulin kinase II pathway [42]. Antagonism of BK channels has been shown to induce TNF-α production [40,43]. Taking into consideration that TNF-α is a major cytokine known to induce cancer cell death through sustained JNK-activation, it can be concluded that the antiproliferative activity of penitrems is associated, in part, with the upregulation of TNF-α and subsequent activation of apoptotic cell death [44,45].

Numerous reports showed the dependence of the cell cycle progression on the translocation of ions across the plasma membrane [13]. The antiproliferative activity of **1** was also shown to be mediated by the induction of G1 cell cycle arrest in BT-474 cells (Figure 11). The epidermal growth factor receptor (EGFR) and the epidermal growth factor receptor 2 (HER2) are ErbB family of receptor tyrosine kinases usually dysregulated in many BC subtypes [46]. Receptor tyrosine kinases (RTKs) are key regulators of cellular growth and proliferation, and dysregulation of RTKs promotes aggressive tumorigenesis pattern [47]. The EGFR/HER2 exert their biological effects through activating multiple downstream signaling cascades, including the PI3K/AKT, ERK1/2, and the transcription protein STAT [48]. Activated K^+^ channel has been shown to increase proliferation rates in several BC cell lines [49,50]. The Ca^2+^-activated K^+^ currents have been observed in multiple BC cell lines, but their role was not clearly understood [34,39].

Small molecule tyrosine kinase inhibitors are increasingly recognized and utilized as treatments targeting specific signaling pathways associated with tumor development and progression. The small molecules lapatinib (LP) is a dual EGFR/HER2 inhibitor, while gefitinib (GF) is a selective EGFR inhibitor approved by FDA for oral control of HER-dependent malignancies, including BC. Despite the success of LP and GF as HER-targeting therapy, limitations to their clinical use involve significant hepatotoxicity and emergence of both primary and acquired resistance [3,4,5]. Many RTKs downstream effects are mediated by calcium signaling, which is regulated by BK channels [51].

Calculated CI of combined **1** with either LP or GF treatments indicated synergistic effects, with CI values less than 1. In addition, isobolograms showed synergistic interactions between **1** and both drugs as indicated by most data points located far below the line of additivity (Figure 12B and Figure 13B). Combination synergy was also associated with reduced levels of p-EGFR, p-HER2, p-AKT, and p-STAT3, which are critical for cell survival and growth. Combinations also enhanced the level of the arrest protein p27. Reduced expression of p27 has also been shown to correlate with the ability of HER2-positive BC cells to escape HER2-targeting therapies, like trastuzumab [55]. The synergistic effect of these combinations is interesting, as it provides the opportunity to reduce the therapeutic doses of the HER family-targeted therapies LP and GF, which would ultimately reduce their potential toxicities and minimize the ability of HER2-dependent BC to develop resistance to targeted therapies.

Collectively, combined treatment of **1** and the anti-HER targeted therapies lapatinib and gefitinib showed remarkable synergistic antiproliferative activity associated with reduced target receptor activation, along with downstream effectors. This clearly highlights the role of BK channel as an appealing molecular target in BC. 

## 4. Materials and Methods

### 4.1. Chemicals

All chemicals were purchased from Sigma-Aldrich (St. Louis, MO, USA), unless otherwise stated. Organic solvents were purchased from VWR (Suwanee, GA, USA), dried by standard procedures, packaged under nitrogen in Sure/Seal bottles, and stored over 4 Å molecular sieves. Unless otherwise indicated, cell culture reagents were obtained from Life Sciences (Carlsbad, CA, USA). Penitrems **1** and **2** were isolated from the marine-derived *Penicillium commune* isolate GS20, while penitrem **3** was semisynthetically prepared from **1** by direct methylation, as described earlier [30,31,49].

### 4.2. Docking Study

In silico binding studies were performed using Schrödinger molecular modeling software (v9, Schrodinger, New York, NY, USA), as previously described [56]. The calcium site domain of each of the BK channel crystal structure PDB codes 3NAF and 3MT5, resolution ≥3 Å, was prepared using the protein preparation wizard, applying PROPKA (Jensen Research Group, Denmark) to add hydrogens, assign bond orders, and optimize hydrogen bonding networks. The optimizing potentials for liquid stimulation (OPLS) force field (OPLS-2005, Schrodinger, New York, NY, USA) were used to ensure proper energy minimization (RMSD = 0.2 Å). The grid generation wizard was then applied on the resulting prepared BK channels structures to create the receptor energy grids as a cubic box centered on the co-crystallized ligand of each crystal structure, and extended for 10 Å in all directions. Penitrem analogs **1**–**3** were drawn using ChemDraw Professional 16 and imported as molfiles to the Maestro 9.3 panel interface (Maestro version 9.3, 2012, Schrodinger, New York, NY, USA). The LigPrep wizard using OPLS force field was chosen to prepare the 3D-dimensional structure of each analog, undergo geometric optimization, search for different conformers, and calculate partial atomic charges. Docking of penitrems was then conducted using the Glide 5.8 module (Glide, 2012, Schrodinger, New York, NY, USA) in extra-precision (XP) mode or in standard-precision mode (SP) [56].

### 4.3. Cell Viability Assay

Penitrems were dissolved in dimethyl sulfoxide (DMSO) to provide a final 25 mM stock solution. Proper media were used to prepare penitrems at their final tested doses for each assay. The DMSO vehicle control was prepared by adding the maximum volume of DMSO used in preparing tested penitrem to appropriate media type, in which the final DMSO concentration did not exceed 0.2% [30,31]. The effects of penitrems **1**–**3** on the proliferation and growth of multiple BC cell lines were evaluated using MTT assay. MDA-MB-231, BT-474, and SK-BR-3 BC cells (ATCC, Manassas, VA, USA) were initially seeded at 1 × 10^4^ cells/well (6 replicates/group) in 96-well plates in RPMI-1640 media containing 10% FBS, and allowed to attach overnight. The next day, cells were washed with PBS, divided into different treatment groups, and then given various concentrations of tested penitrems in 0.5% FBS containing media. Viable cell number was calculated after 48 h using the MTT assay [30].

To evaluate the effect of penitrems on growth of the human neuronal Schwann CRL 2765 (ATCC, Manassas, VA, USA), cells were plated at a density of 1 × 10^4^ cell per well (6-wells/group) in 96-well culture plates using the ATCC-recommended media (Dulbecco’s Modified Eagle’s Medium, DMEM) and allowed to adhere overnight for growth studies, after which each treatment was added, and cells were maintained for 48 h [49]. Similarly, the non-tumorigenic mammary epithelial MCF-12A cells were plated in DMEM/F12 supplemented with 5% horse serum, 0.5 mg/mL hydrocortisone, 20 ng/mL EGF, 100 U/mL penicillin, 0.1 mg/mL streptomycin, and 10 mg/mL insulin. Next day, cells were divided into different groups and fed serum-free DMEM media supplemented with experimental treatments or vehicle-treated control media. Viable cell number was determined using the MTT colorimetric assay. The absorbance was measured at λ_570_ nm on a BioTek Synergy2 microplate reader (BioTek, Winooski, VT, USA). The number of cells per well was calculated against a standard curve prepared at the start of the experiment. The IC_50_ value for each tested sample was calculated by non-linear regression of log concentration versus the % survival, implemented in GraphPad^®^ PRISM version 5.0 (Graph Pad Software, Inc., La Jolla, CA, USA).

### 4.4. Western Blot Analysis

Western blot analysis was used to demonstrate the effects of penitrem treatments on BK channel and TNF-α expression level, and to study the combination effects of penitrem A with LP and GF. MDA-MB-231, BT-474, SK-BR-3, Schwann CRL-2765, and MCF-12A cells, were plated at a density of 1 × 10^6^ cells/100 mm culture plates. MDA-MB-231, BT-474, and SK-BR-3 were plated in RPMI-1640 media supplemented with 10% FBS, Schwann cells CRL-2765 were plated in DMEM media supplemented with 10% FBS [11], and MCF-12A cells were plated in DMEM/F12 supplemented with ingredients described earlier. Cells were allowed to adhere overnight, then washed twice with PBS, and starved in control or treatment in serum-free medium containing 0.5% FBS for 48 h to synchronize the cells in G1 phase. Afterwards, cells were treated with tested penitrems in serum-free defined media for 24 h. At the end of treatment periods, cells were lysed in RIPA buffer (Qiagen Sciences Inc., Valencia, CA, USA). Protein concentration was determined by the BCA assay (Bio-Rad, Hercules, CA, USA). Equivalent amounts of protein (50 µg) were electrophoresed on SDS-polyacrylamide gels. The gels were then electroblotted onto PVDF membranes. These PVDF membranes were then blocked with 2% BSA in 10 mM Tris-HCl containing 50 mM NaCl and 0.1% Tween 20, pH 7.4 (TBST), and then incubated with specific primary antibodies overnight at 4 °C. The following dilutions were used—1:500 for BK channel antibody (Abbiotec LLC., San Diego, CA, USA) and 1:1000 for each of TNF-α, EGFR, pEGFR, STAT, pSTAT, HER2, pHER 2, AKT, pAKT, and p27 (Cell Signaling, Danvers, MA, USA). At the end of incubation period, membranes were washed 5 times with TBST, and then incubated with respective horseradish peroxide-conjugated secondary antibody in 2% BSA in TBST for 1 h at room temperature (rt), followed by rinsing with TBST for 5 times. Blots were then visualized and pictures taken using an enhanced chemiluminescence detection system according to the manufacturer’s instructions (Chemi-Doc^TM^ touch imaging system, Bio-Rad, Hercules, CA, USA). The visualization of β-tubulin was used to ensure equal sample loading in each lane. Western blot quantification analysis was done using Image Lab ^TM^ Software (Bio-Rad, Hercules, CA, USA) for total protein normalization.

### 4.5. Immunocytochemistry

Immunocytochemical fluorescent studies were conducted by growing MDA-MB-231 and BT-474 cells mammary cancer cells on 8-chamber culture slides (Becton Dickinson and Company, Franklin Lakes, NJ, USA) at a density of 2 × 10^4^ cells/chamber (2 replicates/group), and allowed to attach overnight in complete serum media supplemented with 10% FBS. Cells were then washed with Ca^2+^ and Mg^2+^-free phosphate buffered saline (PBS), and incubated with vehicle control or treatment media containing 0.5% FBS for 48 h. At the end of experiment, cells were washed with pre-cooled PBS, and fixed with acetone, pre-cooled to −20 °C, for 2 min. Fixed cells were then washed with PBS, and blocked with 2% BSA in 10 mM Tris-HCl containing 50 mM NaCl and 0.1% Tween 20, pH 7.4 (TBST) for 1 h at rt. Cells were then incubated with specific primary antibody for BK channel KCNMA1 (1:300) overnight at 4 °C in 2% BSA-TBST. At the end of incubation time, cells were washed five times with pre-cooled PBS, followed by incubation with Alexa-Fluor 594-conjugated secondary antibody (1:5000) in 2% BSA-TBST for 1 h at rt. After final washing for five times with PBS, cells were embedded in Vectashield Mounting Medium with DAPI (Vector Laboratories Inc., Burlingame, CA, USA). Fluorescent images were obtained by using a confocal laser scanning microscope (Carl Zeiss Microimaging Inc., Thornwood, NY, USA). The red color indicates the positive fluorescence staining for BK channel KCNMA1, and the blue color represents MDA-MB-231 cell nuclei counter-stained with DAPI [30]. The experiment was repeated at least three times and multiple images for each chamber were captured. Magnification of each photomicrograph is 20×.

### 4.6. Analysis of Cell Cycle Progression by Flow Cytometry

To study **1** effect on cell cycle, BT-474 cells were plated, allowed to synchronize in G_1_ phase, and then treated with **1** at concentrations of 0, 5, 10, 20, and 30 µM. At the end of the experiment, cells in different treatment groups were isolated with trypsin and then resuspended in ice-cold PBS, fixed with cold (−20 °C) 70% ethanol, and stored at 4 °C for 2 h. The cells were then rehydrated with ice-cold PBS and incubated with DNA staining buffer (sodium citrate 1 mg/mL, Triton X-100 3 µL/mL, PI 100 µg/mL, ribonuclease A 20 µg/mL) for 30 min at 4 °C in the dark. DNA content was analyzed using a FACScaliber flow cytometer (BD Biosciences, San Jose, CA, USA). For each sample, 10,000 events were recorded. A histogram was generated using Cell Quest software (BD Biosciences, San Jose, CA, USA). All experiments were repeated at least three times.

### 4.7. Statistical Analysis

The biochemical parameters were analyzed by repeated measures, one-way ANOVA followed by Tukey’s test. Differences with *p* < 0.05 were considered statistically significant.

## 5. Conclusions

**Figure 16:**
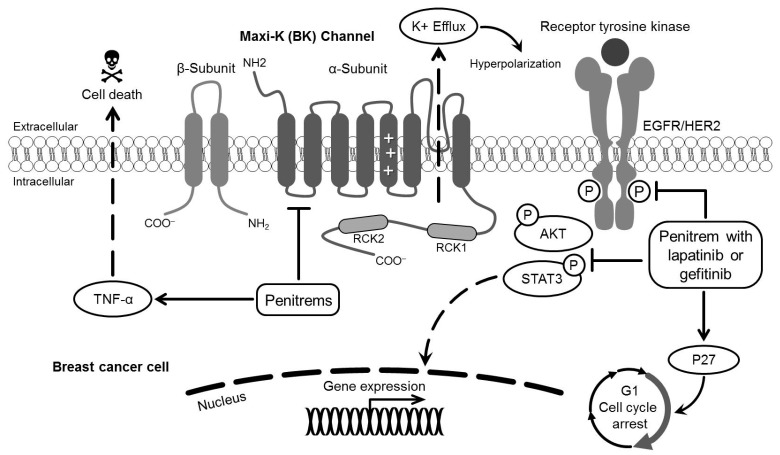
Summary of the pharmacological effects of the BK channel antagonist **1** and its combinations with the HER family-targeting drugs lapatinib and gefitinib in the human luminal B BT-474 BC cells.

Findings from this study revealed remarkable expression of BK channels in multiple BC subtypes, and the potential pharmacological targeting for this channel. Penitrem **1** is a BK channel antagonist with promising antiproliferative activity in BC subtypes overexpressing BK channels. Combination of penitrem A with HER-targeting drugs, LP or GF, resulted in synergistic antiproliferative effects via STAT3 and p27 pathways (Figure 16), which could represent a novel strategy to improve cancer cell sensitivity to targeted regimens and reduce the emergence of resistance to these treatments.

## Figures and Tables

**Table 1:** The IC_50_ (µM) values of penitrems **1**–**3** in multiple breast cancer (BC) and non-cancer cell lines in vitro.

Compound	MCF-12A	CRL-2765	MDA-MB-231	BT-474	SK-BR-3
**1**	33.7	22.6	9.8	10.3	15.1
**2**	44.0	67.8	20.3	31.8	36.7
**3**	78.8	48.2	37.8	22.4	27.1

**Table 2:** Summary of combinations of **1** with lapatinib (LP) or gefitinib (GF) against BT-474 BC cells.

IC_50_/Combination Index (CI) Values				
LP	LP + 1 (0.5 μM)	LP + 1 (1.0 μM)	LP + 1 (2.5 μM)	LP + 1 (5.0 μM)
123.0 nM	65.68 nM/0.58	73.56 nM/0.70	69.80 nM/0.81	66.17 nM/1.03
GF	GF + 1 (0.5 μM)	GF + 1 (1.0 μM)	GF + 1 (2.5 μM)	GF + 1 (5.0 μM)
302.4 nM	124.77 nM/0.46	176.78 nM/0.68	130.84 nM/0.68	114.42 nM/0.87

## Supplementary Materials

The following are available online at http://www.mdpi.com/1660-3397/16/5/157/s1, Figures S1–S3: Additional docking and binding modes of penitrems **2** and **3**.
