# Supplementary material for: The Maxi-K (BK) Channel Antagonist Penitrem A as a Novel Breast Cancer-Targeted Therapeutic
Source: Mar Drugs. 2018 May 11;16(5):157. doi: 10.3390/md16050157 (PMC5983288; doi:10.3390/md16050157)
Supplement: Supplementary file 1 [file marinedrugs-16-00157-s001.pdf]

Article

Supplementary data

The Maxi-K (BK) Channel Antagonist Penitrem A as A Novel Breast Cancer Targeted Therapeutic

Amira A. Goda,[1] Abu Bakar Siddique,[1] Mohamed M. Mohyeldin,[1,3] Nehad M. Ayoub,[2] Khalid A. El Sayed[1]*

[1]*Department of Basic Pharmaceutical Sciences, School of Pharmacy, University of Louisiana at Monroe, Monroe, Louisiana, 71201, USA.*

[2]*Department of Clinical Pharmacy, Faculty of Pharmacy, Jordan University of Science and Technology, Irbid 22110, Jordan.*

[3]*Department of Pharmacognosy, Faculty of Pharmacy, Alexandria University, Alexandria 21521, Egypt.*

*Correspondence: Professor Khalid El Sayed, Department of Basic Pharmaceutical Sciences, School of Pharmacy, University of Louisiana at Monroe, 1800 Bienville Drive, Monroe, Louisiana 71201, USA. Phone: +1-318-342-1725; Fax: +1-318-342-1737; E-mail: elsayed@ulm.edu

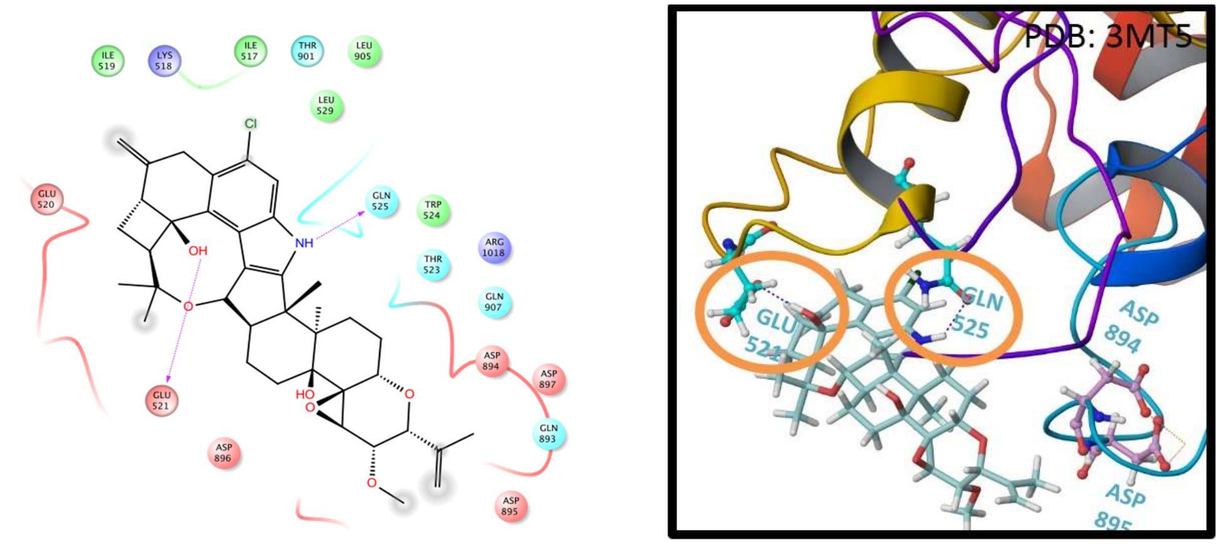

(a) (b)

Figure S1. The 2D binding mode and interactions of **3** at the calcium bowel of the BK channel PDB crystal structure 3MT5. Its C-15 tertiary hydroxyl group contributed hydrogen bonding donor interaction with GLU521 while its NH-1 showed hydrogen bonding donor interaction with GLN525. (b) The overlay of the 3D structure of **3** at the calcium bowel of the BK channel PDB crystal structure 3MT5.

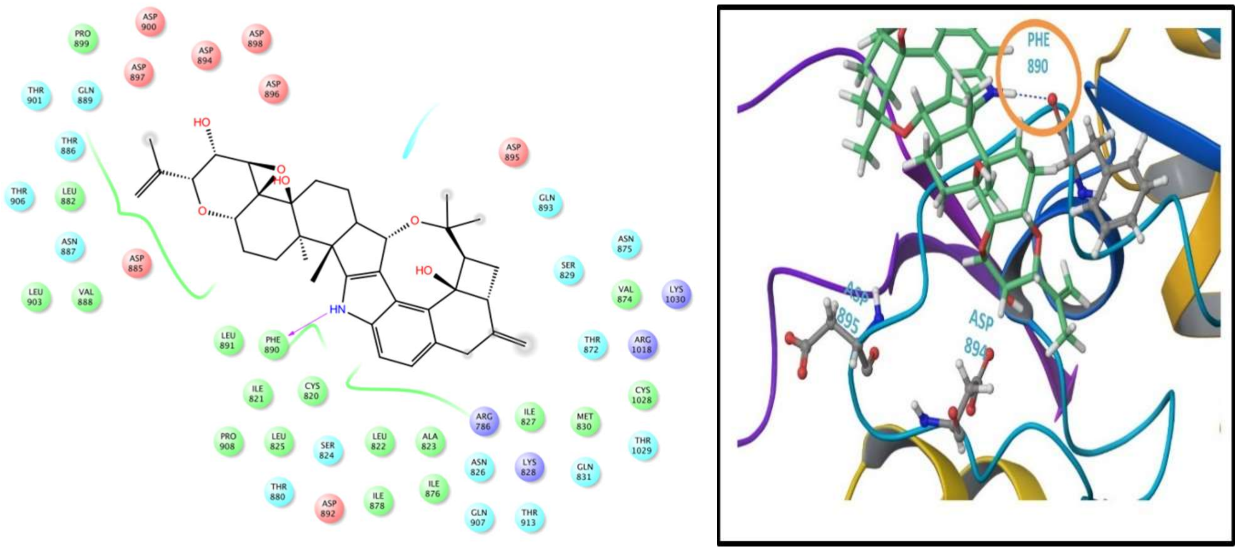

(a) (b)

Figure S2. (a) The 2D binding mode and interactions of **2** at the calcium bowel of the BK channel PDB crystal structure 3NAF. Penitrem E showed only one interaction, its NH-1 contributed hydrogen bonding donor interaction with PHE890. (b) The overlay of 3D structure of **2** at the calcium bowel of the BK channel PDB crystal structure 3NAF.

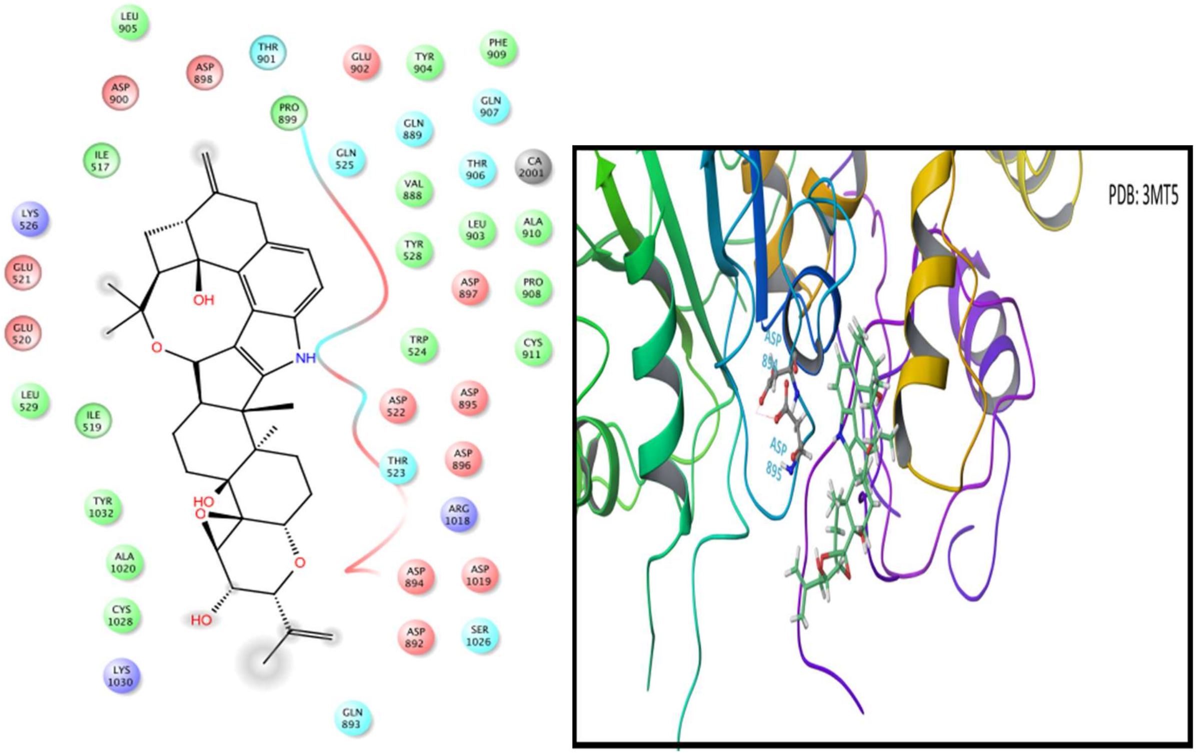

(a) (b)

Figure S3: No interaction of **2** at the calcium bowel of the PDB: 3MT5 crystal structure of the BK channel.
